# Predictive Value of Multiple Scoring Systems in the Prognosis of Septic Patients with Different Infection Sites: Analysis of the Medical Information Mart for the Intensive Care IV Database

**DOI:** 10.3390/biomedicines12071415

**Published:** 2024-06-25

**Authors:** Di Zhang, Changyong Wang, Qianfeng Li, Yi Zhu, Handong Zou, Guang Li, Liying Zhan

**Affiliations:** 1 Department of Critical Care Medicine, Renmin Hospital of Wuhan University, Wuhan 430060, China; zhangdi2005@whu.edu.cn (D.Z.); wcywhu@sina.com (C.W.); zhuyi190@163.com (Y.Z.); zouhandong@whu.edu.cn (H.Z.); 2 Department of Neurosurgery, Wuhan No. 1 Hospital, Wuhan 430022, China; qianfengli2007@126.com

**Keywords:** sepsis, infection site, LODS, OASIS, SOFA, prognosis

## Abstract

The heterogeneity nature of sepsis is significantly impacted by the site of infection. This study aims to explore the predictive value of multiple scoring systems in assessing the prognosis of septic patients across different infection sites. Data for this retrospective cohort study were extracted from the Medical Information Mart for Intensive Care IV database (MIMIC-IV) (v2.2). Adult patients meeting the criteria for sepsis 3.0 and admitted to the intensive care unit (ICU) were enrolled. Infection sites included were pneumonia, urinary tract infection (UTI), cellulitis, abdominal infection, and bacteremia. The primary outcome assessed was 28-day mortality. The sequential Organ Failure Assessment (SOFA) score, Oxford Acute Severity of Illness Score (OASIS), and Logistic Organ Dysfunction System (LODS) score were compared. Binomial logistic regression analysis was conducted to evaluate the association between these variables and mortality. Additionally, differences in the area under the curve (AUC) of receiver operating characteristic (ROC) among the scoring systems were analyzed. A total of 4721 patients were included in the analysis. The average 28-day mortality rate was 9.4%. Significant differences were observed in LODS, OASIS, and SOFA scores between the 28-day survival and non-survival groups across different infection sites (*p* < 0.01). In the pneumonia group and abdominal infection group, both the LODS and OASIS scoring systems emerged as independent risk factors for mortality in septic patients (odds ratio [OR]: 1.165, 95% confidence interval [CI]: 1.109–1.224, *p* < 0.001; OR: 1.047, 95% CI: 1.028–1.065, *p* < 0.001) (OR: 1.200, 95% CI: 1.091–1.319, *p* < 0.001; OR: 1.060, 95% CI: 1.025–1.095, *p* < 0.001). For patients with UTI, the LODS, OASIS, and SOFA scoring systems were identified as independent risk factors for mortality (OR: 1.142, 95% CI: 1.068–1.220, *p* < 0.001; OR: 1.062, 95% CI: 1.037–1.087, *p* < 0.001; OR: 1.146, 95% CI: 1.046–1.255, *p* = 0.004), with the AUC of LODS score and OASIS significantly higher than that of the SOFA score (*p* = 0.006). Among patients with cellulitis, the OASIS and SOFA scoring systems were identified as independent risk factors for mortality (OR: 1.055, 95% CI: 1.007–1.106, *p* = 0.025; OR: 1.187, 95% CI: 1.005–1.403, *p* = 0.044), with no significant difference in prognosis prediction observed (*p* = 0.243). In the bacteremia group, the LODS scoring system was identified as an independent risk factor for mortality (OR: 1.165, 95% CI: 1.109–1.224, *p* < 0.001). The findings suggest that LODS scores offer better prognostic accuracy for predicting the mortality risk in septic patients with pneumonia, abdominal infections, bacteremia, and UTI compared to SOFA scores.

## 1. Introduction

Sepsis is defined as life-threatening organ dysfunction caused by a dysregulated host response to infection. It remains the leading cause of death worldwide [1,2]. Despite advancements in sepsis treatment strategies, leading to a decrease in mortality rates, but it is still at an extremely high level [3,4,5]. The treatment of sepsis necessitates considerable medical and health infrastructures, exerting an appreciable economic encumbrance upon societal resources [6]. The high heterogeneity of sepsis is a significant contributing factor to the challenges in treatment. While the role of the infection site in sepsis remains unclear [7], a prospective study has suggested that it may contribute to the heterogeneity of sepsis and may even be an important factor that should not be ignored [8].

With advancements in medical technology, various scoring systems have been established to evaluate the severity of critically ill patients. The Logistic Organ Dysfunction System (LODS), an organ dysfunction scoring system proposed by Le Gall et al., has also been used to assess organ function in critically ill patients since 1996 [9]. Johnson developed a new reduced severity of illness score using machine-learning algorithms, the Oxford Acute Severity of Illness Score (OASIS), which contained 10 parameters without any laboratory tests and had discrimination and calibration equivalent to more complex existing models [10]. The Sequential Organ Failure Assessment (SOFA), which was initially designed to evaluate the severity of organ dysfunction in patients who were critically ill from sepsis, was introduced for the diagnosis of sepsis [1,11]. All the three scoring systems have proven to be of great significance in predicting the prognosis of septic patients [12,13,14,15,16]. Studies have shown that, compared with patients with pulmonary or intraperitoneal sepsis, patients with primary bacteremia sepsis have a higher mortality rate. It indicates that mortality outcomes can be influenced by the site of infection [17,18]. Consequently, scholars have proposed the development and validation of prognostic models specifically tailored to each infection site [19].

To address these issues, we utilized the Medical Information Mart for Intensive Care (MIMIC)-IV, a comprehensive database for critical care in the United States, to obtain relevant clinical data and explore the predictive value of multiple scoring systems in predicting the prognosis of septic patients across different infection sites.

## 2. Materials and Methods

### 2.1. Data Source

MIMIC-IV is a publicly available database developed by the Massachusetts Institute of Technology Lab for Computational Physiology, containing the information of inpatients at the Beth Israel Deaconess Medical Center from 2008 to 2019. This is a large, single-center, open access database, comprised of details of over 500,000 hospital admissions and 70,000 ICU admissions. It includes information on patient demographics, vital signs, laboratory results, medications, diagnoses, procedures, and other clinical data. Research team members obtained research permission for the MIMIC database (certificate number: 54882354; date: 13 March 2023). All data used in the current analysis were retrieved from the MIMIC-IV 2.2 database. All patient-related information used in this study is anonymized, and no informed consent is required. 

### 2.2. Inclusion and Exclusion Criteria

Adult patients meeting the criteria for sepsis 3.0, admitted to the ICU between 2008 and 2019, were enrolled [1]. Patients with 1 diagnosis code for pneumonia, cellulitis, abdominal infection (including cholecystitis, gastrointestinal perforation, and peritonitis), bacteremia, or urinary tract infection (UTI) were included. Patients meeting any of the following criteria were excluded from the analysis: (I) aged < 18 years; (II) pregnant or breastfeeding women; (III) patients with ICD codes for more than one type of infection; and (IV) missing key rating data.

### 2.3. Data Extraction and Processing

The MIMIC-IV database files were obtained with permission from Physionet, downloaded, and subsequently installed and imported into PostGres 12.0 software. Data retrieval and extraction were performed by establishing a connection using Navicat Premium 15.08 software with Structured Query Language (SQL). Demographic data, Charlson Comorbidity Index (CCI), coexisting comorbidities (including congestive heart disease, myocardial infarction, cerebrovascular disease, renal disease, liver disease, diabetes, and cancer), variables for SOFA score, OASIS, and LODS score calculations, as well as outcomes were extracted from the electronic medical record. The SOFA score, OASIS, and LODS score were calculated based on the worst measurement recorded within the first 24 h of ICU admission. The resulting data were imported into a spreadsheet for analysis.

### 2.4. Grouping

Patients were categorized into the Pneumonia Group, UTI Group, Cellulitis Group, Abdominal Infection Group, and Bacteremia Group based on the different sites of infection. Each group was further divided into the survival group and non-survival group based on the 28-day prognosis.

### 2.5. Statistical Analysis

Descriptive statistics were presented as means with standard deviations, medians with interquartile ranges, or counts with frequencies, depending on the type and distribution of the data. Comparisons between groups were conducted using the Fisher’s exact test *for categorical data*, and the *T* test, Wilcoxon rank-sum test, analysis of variance, or Kruskal–Wallis test for continuous data as appropriate.

Binomial logistic regression analysis was performed to predict 28-day mortality among intensive care patients with sepsis, using age, gender, LODS, OASIS, and SOFA as independent variables. Variables with a *p*-value less than 0.1 in the univariate analysis were included in the multivariate analysis. The discriminatory power of the models was assessed using the AUC ROC. Comparisons between the AUCs were made using the method described by DeLong et al. [20] and analyzed using MedCalc software version 19.1.3. All other statistical analyses were carried out using SPSS 17.0 software, with a *p* value of less than 0.05 considered statistically significant.

## 3. Results

### 3.1. Baseline Data

A total of 4721 cases were included in this study. Baseline characteristics are summarized in Table 1. The median age of the population was 68.7 years, with males comprising 49.0% of the cohort. The predominant infection types were pneumonia (36.3%), UTI (29.9%), and cellulitis (12.7%). Among septic patients, the most prevalent complications were diabetes (37.6%), congestive heart failure (35.3%), and renal disease (34.0%). The average mortality rate at 28 days was 9.4% (444/4721) (Table 1).

### 3.2. Comparison of Scoring Systems in the Survival Group and Non-Survival Group across Infection Sites

Significant differences were observed in LODS, OASIS, and SOFA scores between the 28-day survival and non-survival groups with different infection sites (*p* < 0.01), while gender did not show significant differences in all the groups (*p* > 0.05). Significant differences were observed in age and CCI between the 28-day survival and non-survival groups with pneumonia (*p* < 0.01), which were similar to the results of the abdominal infection group and UTI group. However, there were no significant difference in age and CCI between the 28-day survival and non-survival groups with bacteremia (*p* > 0.05) (Table 2). 

### 3.3. Evaluation of 28-Day Mortality Rates in Septic Patients across Various Infection Sites Using AUC of ROC of Varied Scoring Systems

**Figure 1:**
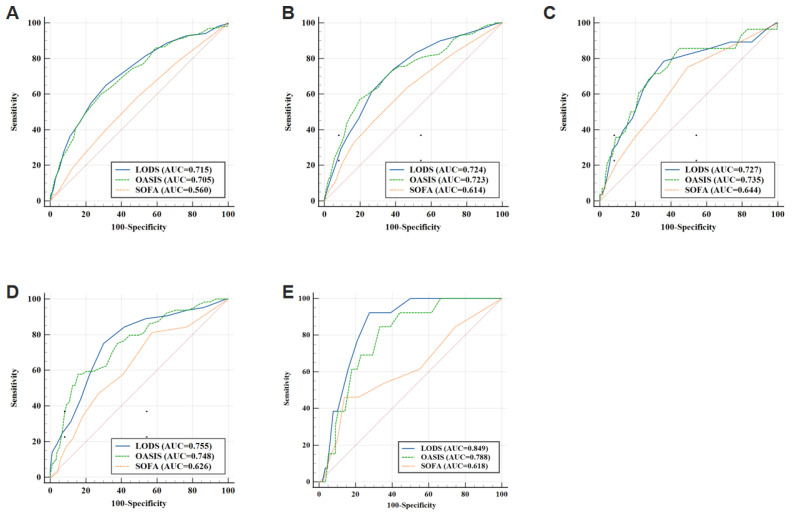
ROC curves of various scoring systems in predicting 28-day mortality rate in septic patients with different infection sites (**A**): Pneumonia Group; (**B**): UTI Group; (**C**): Cellulitis Group; (**D**): Abdominal Infection Group; (**E**): Bacteremia Group. ROC: receiver operating characteristic curve; AUC: area under the curve; LODS: Logistic Organ Dysfunction System; OASIS: Oxford Acute Severity of Illness Score; SOFA: Sequential Organ Failure Assessment.

**Figure 2:**
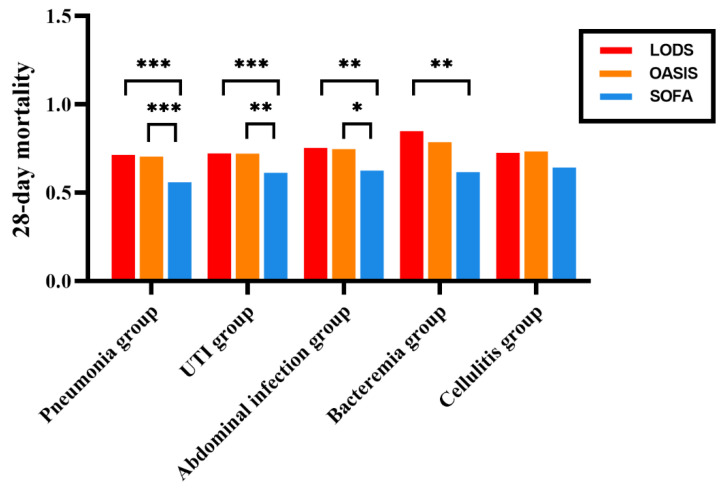
AUROC of the various scoring systems in septic patients with different infection sites. AUROC: area under receiver operator characteristic curve. *, *p* ≤ 0.05; **, *p* ≤ 0.01; ***, *p* ≤ 0.001. LODS: Logistic Organ Dysfunction System; OASIS: Oxford Acute Severity of Illness Score; SOFA: Sequential Organ Failure Assessment; UTI: urinary tract infection.

Pneumonia group: The area under the curves (AUCs) for LODS, OASIS, and SOFA scores were 0.715, 0.705, and 0.560, respectively. Notably, the AUC of LODS scores and OASIS were significantly higher than that of the SOFA score (*p* < 0.001), while there were no significant difference between LODS score and OASIS (*p* = 0.566) (Table 3, Figure 1 and Figure 2).

UTI group: The AUCs for LODS, OASIS, and SOFA scores were 0.724, 0.723, and 0.614, respectively. The AUCs of LODS scores and OASIS were significantly higher than those of the SOFA score (*p* = 0.001; *p* = 0.006), while there was no significant difference between LODS score and OASIS (*p* = 0.990). (Table 3, Figure 1 and Figure 2).

Cellulitis group: The AUCs for LODS, OASIS, and SOFA scores were 0.727, 0.735, and 0.644, respectively. There was no significant difference in AUCs among these three scoring systems (*p* = 0.854; *p* = 0.250; *p* = 0.243) (Table 3, Figure 1 and Figure 2).

Abdominal group: The AUCs for LODS, OASIS, and SOFA scores were 0.755, 0.748 and 0.626, respectively. The AUCs of LODS scores and OASIS were significantly higher than those of the SOFA score (*p* = 0.004; *p* = 0.020), with no significant difference between LODS score and OASIS (*p* = 0.796) (Table 3, Figure 1 and Figure 2). 

Bacteremia group: The AUCs for LODS, OASIS, and SOFA scores were 0.849, 0.788, and 0.618, respectively. The AUC of the LODS score was significantly higher than that of the SOFA score (*p* = 0.008), while there was no significant difference between LODS score and OASIS (*p* = 0.138). There was also no significant difference between OASIS and SOFA scores (*p* = 0.108) (Table 3, Figure 1 and Figure 2).

### 3.4. Binomial Logistic Regression Analysis of Scoring Systems for ICU Mortality in Septic Patients with Different Infection Sites

Single-factor logistic regression analyses revealed that all three scoring systems were significant risk factors for 28-day mortality in septic patients (all *p* < 0.005). 

Multi-factor analysis indicated that the LODS and OASIS scoring systems were independent risk factors for mortality in septic patients with pneumonia or abdominal infection (OR: 1.165, 95% CI: 1.109–1.224, *p* < 0.001; OR: 1.047, 95% CI: 1.028–1.065, *p* < 0.001; OR: 1.200, 95% CI: 1.091–1.319, *p* < 0.001; OR: 1.060, 95% CI: 1.025–1.095, *p* < 0.001). However, there was no significant relationship between the SOFA score and mortality in septic patients in these two groups (OR: 0.739, 95% CI: 0.946–1.081, *p* = 0.739; OR: 1.098, 95% CI: 0.992–1.216, *p* = 0.072) (Table 4).

Multi-factor analysis results show that the OASIS and SOFA scoring systems are independent risk factors for mortality in septic patients with a urinary tract infection or cellulitis (OR: 1.062, 95% CI: 1.037–1.087, *p* < 0.001; OR: 1.146, 95% CI: 1.046–1.255, *p* = 0.004; OR: 1.055, 95% CI: 1.007–1.106, *p* = 0.025; OR: 1.187, 95% CI: 1.005–1.403, *p* = 0.044). The LODS scoring system was also the independent risk factor for mortality in septic patients with a urinary tract infection (OR: 1.142, 95% CI: 1.068–1.220, *p* < 0.001). However, there was no significant relationship between the LODS score and mortality in septic patients with cellulitis. (OR: 1.125, 95% CI: 0.987–1.283, *p* = 0.078) (Table 4).

Multi-factor analysis indicated that the LODS scoring system was an independent risk factor for mortality in septic patients with bacteremia (OR: 1.165, 95% CI: 1.109–1.224, *p* < 0.001). However, there was no significant relationship between the OASIS and mortality in septic patients (OR: 1.061, 95% CI: 0.996–1.131, *p* = 0.066), and similarly, no significant relationship was found between the SOFA score and mortality in septic patients (OR: 1.096, 95% CI: 0.988–1.206, *p* = 0.070) as well (Table 4).

## 4. Discussion

This study aimed to evaluate the prognostic value of various scoring systems in assessing the prognosis of septic patients across different infection sites using a large dataset obtained from public databases. The findings indicate significant associations between the SOFA, LODS, and OASIS scoring systems obtained within 24 h of ICU admission and the 28-day mortality rate among sepsis patients at various infection sites. Among patients with pneumonia, abdominal infection, bacteremia, and urinary tract infection, the LODS scores demonstrated a notably higher predictive capacity for mortality risk compared to SOFA scores. Similarly, OASISs exhibited a significantly higher ability to predict mortality risk than SOFA scores for patients with pneumonia, abdominal infection, and UTI. However, no significant difference was observed in their predictive ability for 28-day mortality in patients with cellulitis.

The SOFA score is an established diagnostic criterion for sepsis 3.0, and is commonly used to evaluate the prognosis of sepsis patients [1]. Prior studies have supported its effectiveness in assessing the prognosis of sepsis cases [21,22,23,24]. Despite its integration into the diagnostic criteria for sepsis 3.0, our findings may suggest that it may not always be the optimal selection to predict the prognosis of sepsis patients, especially those with specific infection sites, including pneumonia, abdominal infection, or bacteremia. Additionally, study indicates that the SOFA score in emergency department patients with sepsis may not be proficient in distinguishing the severity of patients [25]. A comparative investigation discerned that among SOFA, quick sequential Organ Failure Assessment (qSOFA), and conventional assessment metrics, the SOFA score emerged as the most reliable prognostic indicator for sepsis emanating from abdominal infections, yielding an area under the curve (AUC) of 0.889 [26]. This finding diverges from our research, which yielded a lower AUC of 0.626, potentially attributable to the reduced sample size in the aforementioned study.

The OASIS, on the other hand, appears to be more efficacious than the SOFA score in predicting the prognoses of sepsis patients with pneumonia, abdominal infection, and UTI, which is consistent with prior research [12,27]. In a separate investigation, the OASIS was deemed less potent in forecasting ICU and hospital mortality rates among patients with sepsis when contrasted with the SOFA score [21]. The discrepancies in the research outcomes may be attributed to the varied definitions of sepsis, the severity of the condition, and endpoints. Moreover, our findings reveal that the LODS score is superior to the SOFA score for predicting 28-day mortality among septic patients with pneumonia, abdominal infection, bacteremia, and UTI, which is consistent with previous research performed by Zhu et al. [28]. In the other two investigations, the LODS score exhibited a more potent prognostic predictive utility compared to the SOFA score for patients with sepsis, albeit without attaining statistical significance [12,13].

However, there was no significant difference in their predictive ability for 28-day mortality in cellulitis. This highlights the variability in the usefulness of scoring systems in assessing the prognosis of sepsis patients depending on the infection site. In a study by Pawar et al. [29], it was observed that mortality rates, as categorized by quartile of the SOFA score, varied significantly across different infection sites, raising questions about whether sepsis definitions based on scores should be taken into account in the specific context of the infection site. These findings underscore the importance of considering infection site-specific factors when using scoring systems to assess the prognosis of septic patients, thereby informing more tailored and effective management strategies.

This investigation has some limitations. Primarily, it encompasses patients diagnosed with sepsis, all of whom were sourced from the intensive care unit at Beth Israel Medical Center in the United States. As a single-center retrospective study with a predominant focus on the Caucasian population, the potential for racial disparities in the outcomes cannot be overlooked. Furthermore, only septic patients in ICU were considered; we were unable to confirm the same result was applicable to patients with sepsis who were not admitted to the ICU. In addition, National Early Warning Score (NEWS), RAAS (based on the red blood cell distribution, age, Acute Physiology and Chronic Health Evaluation II score, SOFA), and other new scoring systems were not included in this study [30,31,32], and the study is unable to track the dynamic variations within various scoring systems, which could provide a more direct insight into the prognostic indicators for sepsis patients [33]. For a reliable evidence, further multicenter studies are still needed.

## 5. Conclusions

In conclusion, our study highlights the variability in the effectiveness of scoring systems for evaluating the prognosis of septic patients, with outcomes varying according to the specific infection site. Specifically, when predicting 28-day mortality in septic patients with pneumonia, abdominal infection, bacteremia, and UTI, the LODS score emerges as superior to the SOFA score. Meanwhile, in terms of prognostication for septic patients with pneumonia, abdominal infection, and UTI, the OASIS demonstrates greater efficacy compared to the SOFA score.

## Figures and Tables

**Table 1:** Distribution of the baseline characteristics.

	Whole Cohort	Pneumonia Group	UTI Group	Cellulitis Group	Abdominal Infection Group	Bacteremia Group
Total %	4721	1715 (36.3)	1413 (30.0)	597 (12.7)	595 (12.6)	401 (8.5)
Age (year)	68.7 (21.5)	69.2 (21.5)	72.6 (20.8)	65.4 (20.3)	67.0 (20.0)	62.4 (19.6)
Gender *n* (%)						
Male	2315 (49.0)	714 (41.6)	656 (46.4)	349 (58.5)	344 (57.8)	252 (62.8)
Female	2406 (51.0)	1001 (58.4)	757 (53.6)	248 (41.5)	251 (42.2)	149 (37.2)
Ethnicity *n* (%)						
White	3185 (67.5)	1118 (65.2)	964 (68.2)	437 (73.2)	400 (67.2)	266 (66.3)
Black	658 (13.9)	234 (13.6)	208 (14.7)	76 (12.7)	63 (10.6)	77 (19.2)
Others	878 (18.6)	363 (21.2)	241 (17.1)	84 (14.1)	132 (22.2)	58 (14.5)
Comorbidities *n* (%)						
Congestive heart failure	1666 (35.3)	656 (38.3)	532 (37.7)	219 (36.7)	142 (23.9)	117 (29.2)
Myocardial infarct	690 (14.6)	252 (14.7)	231 (16.4)	95 (15.9)	67 (11.3)	45 (11.2)
Cerebrovascular disease	513 (10.9)	192 (11.2)	200 (14.2)	41 (6.9)	47 (7.9)	33 (8.2)
Chronic pulmonary disease	1262 (26.7)	526 (30.7)	383 (27.1)	150 (25.1)	121 (20.3)	82 (20.5)
Renal disease	1605 (34.0)	563 (32.8)	478 (33.8)	242 (40.5)	165 (27.7)	157 (39.2)
Liver disease	1323 (28.0)	432 (25.2)	321 (22.7)	141 (23.6)	297 (49.9)	132 (32.9)
Diabetes	1773 (37.6)	462 (26.9)	607 (43.0)	324 (54.3)	208 (35.0)	172 (42.9)
Cancer	933 (19.8)	358 (20.9)	256 (18.1)	81 (13.6)	156 (26.2)	82 (20.5)
BMI	28.0 (8.8)	27.6 (8.2)	29.1 (10.1)	27.3 (8.6)	27.8 (9.1)	28.5 (9.3)
CRP	29.2 (81.0)	25.1 (85.4)	34.1 (85.7)	23.9 (71.1)	30.7 (74.4)	29.6 (80.25)
28-day mortality *n* (%)	444 (9.4)	220 (12.8)	119 (8.4)	28 (4.7)	64 (10.8)	13 (3.2)

UTI: urinary tract infection. BMI: body mass index; CRP: C-reactive protein.

**Table 2:** The LODS score, OASIS, and SOFA score in septic patients with various infection sites.

	28-Day Survival Group	28-Day Non-Survival Group	*p*-Value
Pneumonia group			
Age (year)	68.5 (21.4)	74.2 (21.6)	<0.01
LODS	6.19 ± 3.38	8.99 ± 3.73	<0.01
OASIS	33.58 ± 9.17	40.80 ± 10.39	<0.01
SOFA	4.03 ± 2.16	4.48 ± 2.37	<0.01
CCI	6.23 ± 2.77	7.03 ± 2.80	<0.01
Gender, male, *n* (%)	885 (59.20)	116 (52.73)	0.069
UTI group			
Age (year)	65.2 (20.1)	74.4 (21.8)	<0.01
LODS	5.97 ± 3.12	8.58 ± 3.31	<0.01
OASIS	33.31 ± 8.84	41.08 ± 9.71	<0.01
SOFA	3.87 ± 1.98	4.71 ± 2.30	<0.01
CCI	6.54 ± 2.60	7.51 ± 2.52	<0.01
Gender, male, *n* (%)	604 (46.68)	52 (43.70)	0.533
Cellulitis group			
Age (year)	65.2 (20.1)	74.4 (21.8)	<0.01
LODS	5.79 ± 3.23	8.64 ± 3.81	<0.01
OASIS	31.53 ± 8.99	39.75 ± 10.76	<0.01
SOFA	3.97 ± 2.06	5.07 ± 2.48	<0.01
CCI	6.27 ± 2.88	6.28 ± 2.26	0.976
Gender, male, *n* (%)	330 (58.0)	19 (67.86)	0.301
Abdominal infection group			
Age (year)	66.5 (19.4)	70.9 (24.5)	0.04
LODS	6.13 ± 3.27	9.44 ± 3.68	<0.01
OASIS	33.17 ± 9.37	42.33 ± 10.07	<0.01
SOFA	4.55 ± 2.51	5.58 ± 2.59	<0.01
CCI	6.09 ± 2.75	6.92 ± 2.90	0.023
Gender, male, *n* (%)	312 (58.76)	32 (50.0)	0.18
Bacteremia group			
Age (year)	62.3 (19.6)	65.9 (27.7)	0.9
LODS	5.98 ± 3.38	10.15 ± 2.19	<0.01
OASIS	32.12 ± 8.97	40.38 ± 6.12	<0.01
SOFA	4.26 ± 2.29	5.38 ± 2.75	<0.01
CCI	5.89 ± 2.73	6.0 ± 3.85	0.923
Gender, male, *n* (%)	244 (62.89)	8 (61.54)	0.921

LODS: Logistic Organ Dysfunction System; OASIS: Oxford Acute Severity of Illness Score; SOFA: Sequential Organ Failure Assessment; CCI: Charlson Comorbidity Index; UTI: urinary tract infection.

**Table 3:** AUCs of various scoring systems in predicting the 28-day mortality rates in septic patients with various infection sites.

Group	Scoring System	AUC	95%	Optimal Cut-Off	Sensitivity	Specificity	Youden Index
Pneumonia group	LODS	0.715	0.692–0.736	7	65.0	68.6	0.336
	OASIS	0.705	0.682–0.726	38	60.0	71.4	0.314
	SOFA	0.560	0.536–0.584	3	58.2	51.1	0.093
UTI group	LODS	0.724	0.70–0.747	6	73.9	61.3	0.352
	OASIS	0.723	0.699–0.747	40	57.1	80.1	0.372
	SOFA	0.614	0.589–0.640	3	63.9	53.2	0.170
Cellulitis group	LODS	0.727	0.689–0.762	6	78.6	64	0.425
	OASIS	0.735	0.698–0.770	31	85.7	55.4	0.411
	SOFA	0.644	0.604–0.683	3	75.0	50.6	0.256
Abdominal infection group	LODS	0.755	0.718–0.789	7	75.0	70.2	0.451
	OASIS	0.748	0.711–0.782	42	57.8	84.2	0.420
	SOFA	0.626	0.586–0.665	3	81.2	42.7	0.240
Bacteremia group	LODS	0.849	0.810–0.833	7	92.3	72.4	0.647
	OASIS	0.788	0.745–0.827	35	84.6	66.8	0.514
	SOFA	0.618	0.569–0.666	6	46.2	86.1	0.322

AUC: area under the curve; UTI: urinary tract infection.

**Table 4:** Binomial logistic regression analysis of the scoring systems for ICU mortality in septic patients with different infection sites.

	Univariable		Multivariable	
	OR (95%CI)	*p*	OR (95%CI)	*p*
Pneumonia group				
Age (year)	1.024 (1.014–1.034)	<0.001	1.017 (1.004–1.029)	0.008
LODS	1.235 (1.186–1.286)	<0.001	1.165 (1.109–1.224)	<0.001
OASIS	1.079 (1.063–1.095)	<0.001	1.047 (1.028–1.065)	<0.001
SOFA	1.090 (1.028–1.156)	0.004	1.011 (0.946–1.081)	0.739
CCI	1.109 (1.054–1.166)	0.018	1.077 (1.013–1.145)	0.018
UTI group				
Age (year)	1.038 (1.022–1.054)	<0.001	1.032 (1.014–1.051)	<0.001
LODS	1.258 (1.190–1.330)	<0.001	1.142 (1.068–1.220)	<0.001
OASIS	1.092 (1.070–1.115)	<0.001	1.062 (1.037–1.087)	<0.001
SOFA	1.186 (1.096–1.282)	<0.001	1.146 (1.046–1.255)	0.004
CCI	1.151 (1.072–1.236)	<0.001	1.096 (1.007–1.192)	0.033
Cellulitis group				
Age (year)	1.045 (1.015–1.076)	0.03	1.043 (1.010–1.077)	0.010
LODS	1.251 (1.128–1.389)	<0.001	1.125 (0.987–1.283)	0.078
OASIS	1.090 (1.049–1.133)	<0.001	1.055 (1.007–1.106)	0.025
SOFA	1.214 (1.052–1.402)	0.008	1.187 (1.005–1.403)	0.044
CCI	1.002 (0.877–1.145)	0.976		
Abdominal infection group				
Age (year)	1.020 (1.001–1.039)	0.041	1.012 (0.988–1.036)	0.323
LODS	1.303 (1.206–1.409)	<0.001	1.200 (1.091–1.319)	<0.001
OASIS	1.097 (1.067–1.127)	<0.001	1.060 (1.025–1.095)	<0.001
SOFA	1.150 (1.050–1.259)	0.003	1.098 (0.992–1.216)	0.072
CCI	1.112 (1.014–1.219)	0.023	1.053 (0.941–1.179)	0.369
Bacteremia group				
Age (year)	1.002 (0.966–1.040)	0.900		
LODS	1.361 (1.167–1.587)	<0.001	1.303 (1.012–1.539)	0.002
OASIS	1.093 (1.034–1.157)	0.002	1.061 (0.996–1.131)	0.066
SOFA	1.149 (1.048–1.229)	0.004	1.096 (0.988–1.206)	0.070
CCI	1.014 (0.831–1.237)	0.892		

LODS: Logistic Organ Dysfunction System; OASIS: Oxford Acute Severity of Illness Score; SOFA: Sequential Organ Failure Assessment; CCI: Charlson Comorbidity Index; UTI: urinary tract infection; OR: odds ratio; CI: confidence interval.

## Data Availability

Data are available on request to the correspondence author.
